# Impact of age, physiological status and APACHE score on acceptance of patients to the ICU

**DOI:** 10.1186/cc14602

**Published:** 2015-03-16

**Authors:** L Terry, S Passey, D Porter, F Clark, R Matsa

**Affiliations:** 1 Royal Stoke University Hospital, Stoke on Trent, UK

## Introduction

Evidence suggests that age, chronic health status and acute illness severity affect the decision-making of clinicians regarding admission to the ICU (ITU) [1-3]. This prospective service review assesses the impact of age, APACHE II score and WHO functional score towards admission acceptance or refusal to ITU in a tertiary-level facility.

## Methods

Design: a planned prospective review of all referrals over a 14-day period. Data collection: review (LT, DP, SP) of case notes of patients referred to ITU with the following variables collected: age, sex, APACHE II scores, WHO functional status score, grade of referrer and source of referral. Data were collected on 37 patients: 22 accepted to ITU and 15 refused admission. Statistics: data were analyzed using GraphPad 6.05. Categorical variables were expressed as mean and standard error of mean. For unpaired variables, statistical significance is determined using unpaired *t *test. *P *< 0.05 is considered statistically significant.

## Results

The WHO functional status was the most significant variable affecting admission (*P *< 0.001). The APACHE score of patients admitted to ITU was significantly lower than refused patients (*P *= 0.039). Patient age did not affect admission status (*P *= 0.15). See Table 1.

**Table 1:** 

	Accepted	Refused	*P *value
Mean age	56.5 ± 4.4	65.3 ± 3.6	0.15
Sex	73% male	56% male	N/A
WHO	0.45 ± 0.2	2.21 ± 0.3	<0.001
APACHE	13.5 ± 1.6	18.8 ± 2.0	0.039

## Conclusion

This study was performed to assess decision-making for admission to the ITU in times of increased demand and limited availability. We want to validate our findings from this short pilot study in a larger prospective study. Multivariate regression analysis will be used to identify significant features that affect clinician decision making in critical care admission.
